# A parametric study of fear generalization to faces and non-face objects: relationship to discrimination thresholds

**DOI:** 10.3389/fnhum.2014.00624

**Published:** 2014-09-05

**Authors:** Daphne J. Holt, Emily A. Boeke, Rick P. F. Wolthusen, Shahin Nasr, Mohammed R. Milad, Roger B. H. Tootell

**Affiliations:** ^1^The Department of Psychiatry, Massachusetts General HospitalBoston, MA, USA; ^2^Harvard Medical SchoolBoston, MA, USA; ^3^The Athinoula A. Martinos Center for Biomedical ImagingCharlestown, MA, USA; ^4^Department of Child and Adolescent Psychiatry, Faculty of Medicine Carl Gustav Carus of the Technische Universität DresdenDresden, Germany; ^5^The Department of Radiology, Massachusetts General HospitalBoston, MA, USA

**Keywords:** fear, faces, emotion, learning, generalization, perception

## Abstract

Fear generalization is the production of fear responses to a stimulus that is similar—but not identical—to a threatening stimulus. Although prior studies have found that fear generalization magnitudes are qualitatively related to the degree of perceptual similarity to the threatening stimulus, the precise relationship between these two functions has not been measured systematically. Also, it remains unknown whether fear generalization mechanisms differ for social and non-social information. To examine these questions, we measured perceptual discrimination and fear generalization in the same subjects, using images of human faces and non-face control stimuli (“blobs”) that were perceptually matched to the faces. First, each subject’s ability to discriminate between pairs of faces or blobs was measured. Each subject then underwent a Pavlovian fear conditioning procedure, in which each of the paired conditioned stimuli (CS) were either followed (CS+) or not followed (CS−) by a shock. Skin conductance responses (SCRs) were also measured. Subjects were then presented with the CS+, CS− and five levels of a CS+-to-CS− morph continuum between the paired stimuli, which were identified based on individual discrimination thresholds. Finally, subjects rated the likelihood that each stimulus had been followed by a shock. Subjects showed both autonomic (SCR-based) and conscious (ratings-based) fear responses to morphs that they could not discriminate from the CS+ (generalization). For both faces and non-face objects, fear generalization was not found above discrimination thresholds. However, subjects exhibited greater fear generalization in the shock likelihood ratings compared to the SCRs, particularly for faces. These findings reveal that autonomic threat detection mechanisms in humans are highly sensitive to small perceptual differences between stimuli. Also, the conscious evaluation of threat shows broader generalization than autonomic responses, biased towards labeling a stimulus as threatening.

## Introduction

Fear generalization is an adaptive process in which a fear response occurs to stimuli that are similar to a threatening stimulus (Lissek et al., 2008; Hajcak et al., 2009; Dunsmoor and Labar, 2013; Haddad et al., 2013). Some generalization of fear responses is presumably crucial for survival, because similar stimuli may well be similarly dangerous. For instance, it is appropriate to be afraid of a dog that looks and sounds like a dog that previously bit you (“once bitten, twice shy”). However, fear generalization processes may be abnormal in some psychopathological states (Lissek, 2012).

The process of stimulus generalization has been studied for decades, using a variety of methods and stimuli, in a range of species including pigeons, goldfish, worms and humans (Ghirlanda and Enquist, 2003). In humans, the generalization of fear-related responses has been studied primarily using Pavlovian fear conditioning paradigms. In these studies, a variety of outcomes have been used to index fear generalization, including electromyography-measured startle responses (Lissek et al., 2008; Hajcak et al., 2009; Haddad et al., 2013), skin conductance responses (SCRs; Vervliet et al., 2010b; Dunsmoor and Labar, 2013) and explicit ratings (ERs) of fear or shock likelihood (Vervliet et al., 2006, 2010b; Lissek et al., 2008; Hajcak et al., 2009). Each of these studies found significantly increased fear-related responses to stimuli that were perceptually similar (compared to those that were less similar) to a conditioned stimulus (CS) that had been paired with an aversive outcome, such as an electrical shock. In other words, conditioned fear responses were found to generalize from a CS (typically an abstract shape such as a circle or a rectangle) paired with a shock (a CS+), compared to a slightly altered version of that CS that was not paired with the shock (a generalization stimulus, GS). Although these studies have described a qualitative association between fear generalization magnitudes and the degree of perceptual similarity of the GSs to the CS+, the precise relationship between discrimination ability and fear generalization in humans has not been systematically studied. One might predict that autonomic measures of fear responses would generalize beyond perceptual discrimination thresholds, i.e., subjects show fear responses to similar but easily distinguishable stimuli. Alternatively, autonomic responses might be more sensitive than perception in some cases, based on prior demonstrations of sub-threshold summation (Kulikowski and King-Smith, 1973; To et al., 2011) and “unconscious” fear responses (Morris et al., 1998; Whalen et al., 1998).

An additional possibility is that fear generalization gradients might narrow or broaden depending on the context or type of stimuli encountered. For example, the ability to both discriminate and extract common features from similar stimuli is important in social contexts. It is often necessary to quickly assess whether an individual is a friend or foe, generalizing from prior experience and erring on the side of a defensive posture when in doubt, until additional information becomes available. However, the benefits of generalization during social interactions are balanced against the advantages of being able to discriminate among specific individuals with whom one has different relationships.

Recognition and discrimination among distinct humans occurs primarily via recognition of faces (McKone et al., 2007). Many lines of evidence suggest that faces are processed in a specialized manner by the brain. For example, psychophysical studies have shown that faces are processed “holistically” (Kemp et al., 1996; Farah et al., 1998; Hole et al., 1999). In contrast, other types of stimuli are processed in a more piecemeal manner, based on their feature components. Face-specific processing mechanisms are anatomically segregated in specialized pathways in the brain in both humans and monkeys (Kanwisher et al., 1997; Tsao et al., 2008; Pinsk et al., 2009; Rajimehr et al., 2009; Ku et al., 2011; Nasr and Tootell, 2012). Thus, it is possible that these unique aspects of face perception influence the generalization of fear responses across perceptually similar faces.

Thus, in the current study, we aimed to (1) measure the relationship between visual discriminability and fear generalization; and (2) compare fear generalization gradients for faces and non-face control stimuli. First, we predicted that significant fear generalization would occur to stimuli that were indistinguishable from a threatening stimulus (one that had been associatively linked to an aversive experience, an electrical shock). Second, we predicted that fear generalization would be greater to faces, compared to non-face control stimuli.

## Materials and methods

### Overview of Experiment 1 (faces) and Experiment 2 (“blobs”)

**Figure 1:**
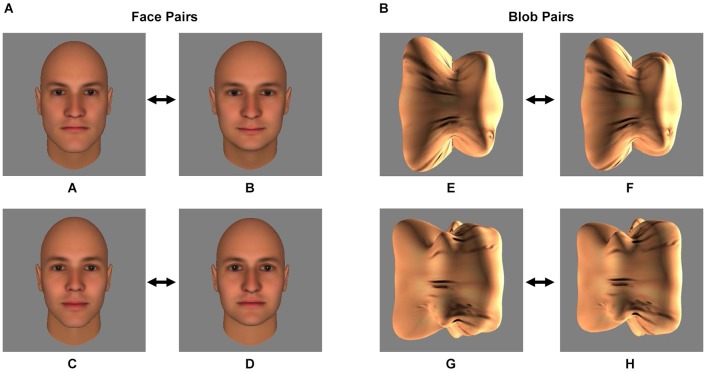
**Stimuli**. The two pairs of face stimuli (A/B and C/D) **(A)** and the two pairs of blob stimuli (E/F and G/H) **(B)** that served as the conditioned stimuli (CS) during the Pavlovian fear conditioning procedure are shown. During the procedure, one of the two stimuli of the pair was followed by an electrical shock (the CS+) and one was not (the CS−). Prior to Pavlovian fear conditioning, each subject’s discrimination ability was measured using a forced-choice discrimination task, in which the CS+ stimulus was displayed next to morphs that were perceptually similar to the CS+ (see Materials and Methods and Figure 2).

We created image morphs between images of (1) two distinct human faces (Experiment 1); and (2) two distinct non-face shapes or “blobs” (Experiment 2) (Figure 1). Later in the experiment, one of the two faces or blobs (the conditioned stimuli, CS) was paired with an electrical shock (the CS+) during a Pavlovian fear conditioning procedure.

**Figure 2:**
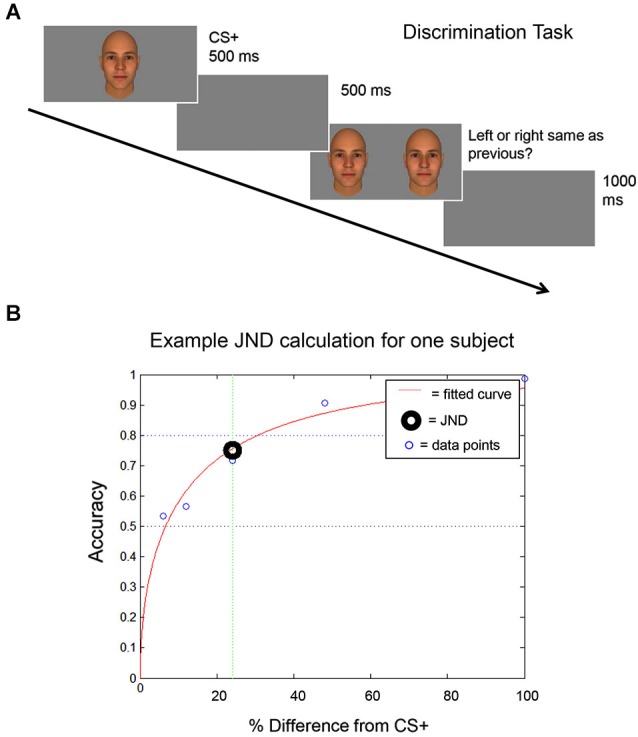
**Discrimination task**. **(A)** A schematic of the discrimination task used to identify each subject’s discrimination threshold (the Just Noticeable Difference (JND) level) is shown. The CS+ stimulus is shown and then, following a 500 ms inter-trial interval (ITI), the CS+ stimulus is shown next to a morph stimulus. **(B)** An example is shown of the curve that is generated using each subject’s performance on the discrimination task, to calculate each subject’s JND. The JND was then used to select the morph stimuli that were presented to that subject during the fear generalization procedure (see Materials and Methods).

First, each subject performed a *discrimination task* to identify the image morph that he could distinguish from the CS+ stimulus at a 75% accuracy level. This value was defined as the Just Noticeable Difference (JND) threshold.

Second, subjects underwent a Pavlovian *fear conditioning procedure*, in which the CS+ stimulus was intermittently followed by a shock, and the other stimulus of the pair was not followed by a shock (the CS−). SCRs were measured continuously.

Third, subjects underwent a *fear generalization procedure* during which they were presented with the CS+, CS− and five morphs whose degree of difference from the CS+ was determined by the subject’s performance on the discrimination task (i.e., the specific JND for that subject). SCRs were measured continuously.

Fourth, subjects were presented with each of the previously presented stimuli, and asked to rate the likelihood that each stimulus had ever been followed by a shock (*explicit ratings*).

### Participants

Seventy-one healthy male volunteers (mean age: 24.61 ± 0.91) were recruited using an on-line advertisement and enrolled in the study (39 and 32 for Experiment 1 and 2, respectively). Only males were included in this initial study in order to minimize SCR heterogeneity related to gender differences in fear responses (Milad et al., 2006). Participants had no history of psychiatric or neurologic illness, as determined by a phone screen and the Structured Clinical Interview for DSM-IV (SCID; First et al., 1995). All subjects had normal or near normal vision, based on Snellen acuity.

The study was approved by the Partners Healthcare Institutional Review Board, and written informed consent was obtained from all subjects at the time of enrollment.

### Stimuli

#### Experiment 1: Faces

Four images of human faces (see Figure 1A) were generated using FaceGen 3.4 (Singular Inversions, Canada), as described previously (Yue et al., 2011, 2013; Holt et al., 2014). All four faces (A, B, C and D) were male and caucasian, and achromatic (i.e., all color parameters were set to 0). FaceGen was then used to create morphs (99 even steps) between faces A and B and between faces C and D.

#### Experiment 2: Non-face “blobs”

Four images of three-dimensional, unfamiliar shapes (“blobs”; see Figure 1B) were generated as described elsewhere (Yue et al., 2013). To equate the texture pattern of the blob and face stimuli, a synthesized texture was generated from scrambling the texture of the face images (Portilla et al., 2003) and overlaid onto the blob stimuli. As with the faces, morphs (99 even steps) were created between blobs E and F and between blobs G and H.

### Discrimination task

Participants were assigned one of the two pairs of face (A/B or C/D, Experiment 1) or blob (E/F or G/H, Experiment 2) stimuli. The assigned face or blob pair was counterbalanced across subjects. Later in the experiment (during the Fear Conditioning procedure, see below), one of the two stimuli (the CS+) was paired with an electrical shock (the unconditioned stimulus, US), while the other stimulus (the CS−) was not paired with a shock. The CS+ and CS− assignment within each pair was counterbalanced across subjects.

Before the Fear Conditioning procedure, the subjects’ ability to discriminate between the pair of stimuli assigned to them was evaluated using a forced-choice discrimination task. Prior to any measurements, the subject practiced the task until they confirmed that they understood the procedure (3–5 trials). The task consisted of three runs of 50 trials each. During each trial, participants first viewed the CS+ stimulus for 500 ms. Following an inter-trial interval (ITI) of 500 ms, the participants were presented with a morph and the CS+ stimulus side by side. Subjects were then asked to select which stimulus they had previously seen, by pressing one of two buttons, indicating the image on the right or the left. The positioning of the morph and CS+ stimulus was randomized across trials. Participants had unlimited time to respond (self-paced). The morphs used in the discrimination task were 6%, 12%, 24%, 48%, and 100% different from the CS+ stimulus (100% different = the other stimulus of the pair, the CS−). The participant’s response was followed by an ITI of 1 s. Following completion of the task, participant accuracy was plotted against the morph level (the percentage difference from the CS+ stimulus), in order to calculate the JND level (Figure 2) for that participant. The JND was the morph level (% difference from the CS+ stimulus, which could fall between the morphs presented during the discrimination task) that could be distinguished from the CS+ stimulus at an accuracy of 75%. For an independent experiment (not shown here), subjects performed this task a second time (3 additional runs) following the completion of the Fear Conditioning and Fear Generalization phases of the experiment.

### Fear conditioning and fear generalization

The Coulbourn Instruments Lablink V System (Allentown, Pennsylvania) was used for these two phases of the experiment. Skin conductance levels were measured with the Coulbourn Isolated Skin Conductance Coupler. Before the Fear Conditioning procedure, two electrodes were placed on the palm of the participant’s left hand (to record SCRs) and on the index finger and middle finger of the participant’s right hand (to deliver the US, a mild electrical stimulus 500 ms in duration). Next, the intensity of the US was set by each participant to a level that was “highly annoying but not painful” (Milad et al., 2005; Holt et al., 2009). Also, prior to these procedures, the subjects were told that, during the experiment, each stimulus may or may not be followed by the US, but one stimulus was more likely to be followed by the US. They were also told that they would be asked questions about what they had observed following the experiment. Throughout these two procedures, subjects were observed through a closed circuit video camera to ensure that they were awake and attentive.

#### Fear conditioning

This phase consisted of 8 CS+ trials and 8 CS− trials, each 6 s long, presented in a pseudorandom order (Milad et al., 2005). ITIs were 9, 12, or 15 s in duration. The CS+ was followed by the US in 5 of the 8 CS+ trials, and the CS− was never followed by a shock.

In the first 17 participants, a pilot version of the Fear Conditioning phase was used (12 trials, 50% reinforcement). Because this version did not produce reliable learning in this group (learning occurred in 12/17 subjects), this phase was modified. (Since the goal of the study was to examine generalization of previously learned fear responses, it was important that subjects demonstrate adequate fear learning initially, see below).

#### Fear generalization

**Figure 3:**
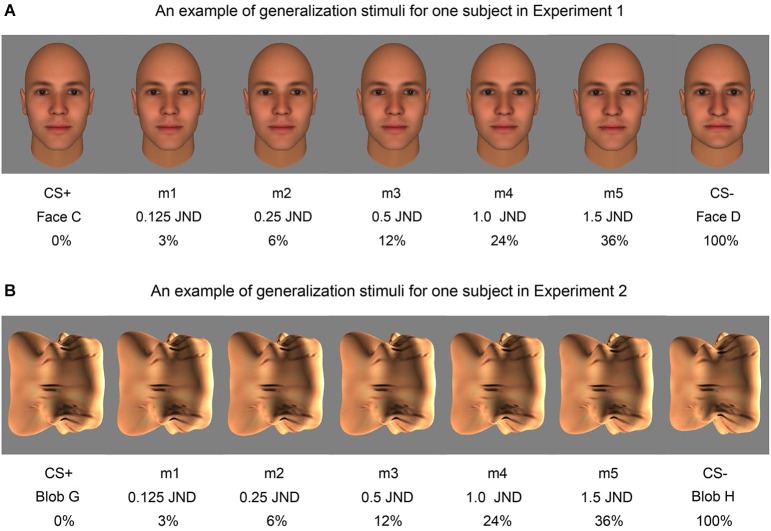
**Examples of the morph stimuli used in fear generalization**. An example of the face morph stimuli shown to one subject in Experiment 1 **(A)** and an example of the blob morph stimuli shown to one subject in Experiment 2 **(B)** are displayed. The percentage difference from the CS+ stimulus (Face C and Blob G in these examples) and the fraction of the JND are both included below the corresponding morph (m1, m2, m3, m4, m5).

This phase began after a 1-min break following Fear Conditioning. During the Fear Generalization procedure, subjects were presented with the CS+, CS− and five morph levels (m1, m2, m3, m4, and m5) whose degree of difference from the CS+ was determined by the subject’s performance on the discrimination task (*m1* = 0.125 JND; *m2* = 0.25 JND; *m3* = 0.5 JND; *m4* = 1.0 JND; *m5* = 1.5 JND) (Figure 3). This phase consisted of 35 trials, i.e., five trials for each stimulus category. The ITIs were again 9, 12, and 15 s in length and each stimulus was presented for 6 s. For each subject, stimuli were presented in one of two different pseudorandom orders, so that no more than two of the same stimuli were presented consecutively (to avoid habituation of responses due to repetition) (Lissek et al., 2008; Dunsmoor et al., 2009), counterbalanced across subjects. During this phase, the CS+ was always followed by the shock (100% reinforcement), in order to minimize extinction of the association produced by viewing many CS+-like stimuli that were not followed by a shock.

### Explicit fear ratings

Following Fear Generalization, subjects were presented with each of the previously presented stimuli once (in one of two pseudorandom orders, counterbalanced across subjects; stimulus presentation time = 6 s; ITI = 9 s), then asked to rate the likelihood that the stimulus had ever been followed by a shock (on a scale of 0–100% likely).

### Skin conductance data pre-processing

During Fear Conditioning and Fear Generalization, skin conductance was recorded continuously. A participant was considered a “responder” if ≥2 of the 16 trials of the Fear Conditioning phase showed a response greater than 0.05 μS (Schnur et al., 1999; Turner et al., 2005). Data from subjects which did not meet this criteria (“non-responders”) were excluded from further analysis (see below).

For both Fear Conditioning and Fear Generalization, the SCR to the stimulus was calculated by subtracting the mean skin conductance for the 2 s prior to stimulus onset from the peak of the skin conductance during the 6 s of stimulus presentation. In addition, for the analysis of the Fear Generalization data only, SCRs were calculated in an identical manner (using the 2 s prior to stimulus onset as the baseline) for the first 6 s of the ITI that immediately followed stimulus offset. Thus, we tested for fear generalization during two time intervals: (1) during the stimulus presentation (*immediate* fear generalization, IFG); and (2) following stimulus offset (*delayed* fear generalization, DFG). SCRs were square-root transformed and averaged across each stimulus type for both Fear Conditioning (CS+ and CS−) and Fear Generalization (CS+, m1, m2, m3, m4, m5, CS−), prior to the statistical analyses.

### Participants included in the analyses

Because our goal was to measure fear generalization in participants who had successfully learned to discriminate the CS+ and CS−, we included data in our analyses from the participants who demonstrated successful learning only. The “learner” criterion for each individual consisted of a difference in shock likelihood ratings between the CS+ and CS− stimuli ≥50%.

Also, data from three subjects were excluded from the analyses because their discrimination task data were unusable; another subject’s data were excluded because he fell asleep during the Fear Generalization procedure. Of the remaining 67 subjects, 53 were learners (28 and 25 subjects in Experiment 1 and 2, respectively). Of the 53 learners, 6 were non-responders (4 and 2 subjects in Experiment 1 and 2, respectively). Thus, 47 subjects (24 and 23 subjects in Experiment 1 and 2, respectively) were included in the analyses (mean age: 23.61 ± 0.94).

Lastly, two of the subjects who participated in Experiment 2 had JND values following the discrimination task that were too high to permit assignment of stimuli. These subjects were assigned generalization stimuli that differed maximally from the CS+ (*m1* = 8%, *m2* = 17%, *m3* = 33%, *m4* = 66%, *m5* = 99%). Excluding these two subjects from the analyses did not alter the findings.

### Data analyses

#### Fear conditioning

The presence of significant differential fear conditioning (CS+ minus CS− responses, *p* < 0.05) was assessed using paired, two-tailed *t*-tests.

#### Fear generalization

In the *SCR data*, we tested for fear generalization using a repeated measures ANOVA with three factors: stimulus level (6: m1, m2, m3, m4, m5, CS−), experimental phase (2: during stimulus presentation, following stimulus offset), and stimulus type (2: faces, blobs) as a between-subjects factor. SCR data collected for the CS+ was not included in this analysis (thus, there are six stimulus levels in this ANOVA), since the presence of the shock during the ITI phase confounds the measurement of the CS+ response following stimulus offset. Significant main effects and interactions with stimulus level (*p* < 0.05) were followed up by paired, two-tailed *t*-tests.

In the *ER data*, we conducted a second repeated measures ANOVA with two factors: stimulus level (7: CS+, m1, m2, m3, m4, m5, CS−), and stimulus type (2: faces, blobs) as a between-subjects factor. Significant main effects and interactions with stimulus level (*p* < 0.05) were followed up by paired, two-tailed *t*-tests.

*Comparison of autonomic and explicit fear generalization*: we compared the amount of fear generalization in the SCRs (independently for the IFG and DFG responses) to the fear generalization in the ERs, using ANOVAs performed on normalized data (normalization permitted comparison of SCR and ratings data). These two ANOVAs included three factors: response type (2: SCR, ratings), stimulus level (seven or six for the IFG and DFG analyses, respectively), and stimulus type (2: faces, blobs) as a between-subjects factor.

For each subject, each averaged value for a given stimulus was normalized using the following formula: (Value − Minimum)/(Maximum − Minimum), where Minimum is the smallest average value for a given subject (i.e., the subject’s average value for the CS+, m1, m2, m3, m4, m5, or CS−, whichever is the smallest), and Maximum is the largest averaged value for the subject (i.e., their average value for the CS+, m1, m2, m3, m4, m5, or CS−, whichever is the largest). Thus, each subject’s largest average response was scaled to 1, and the smallest response was scaled to 0.

#### Correlations

Correlations among fear conditioning, fear generalization (for the morphs to which there was significant fear generalization, see below) and JND levels were examined using Pearsons *r*.

## Results

### Fear conditioning

In both Experiment 1 and 2, subjects acquired differential, conditioned fear responses (CS+ > CS−, *p*s < 0.001). We found no difference between the level of differential fear conditioning acquired during the two experiments (*t*_(45)_ = 0.69, *p* = 0.50; mean SCR to CS+ = 0.40 ± 0.07 μS (mean ± SEM); mean SCR to CS− = 0.16 ± 0.08 μS across all subjects (*n* = 47); comparison of the CS+ vs. CS−: *t*_(46)_ = 7.22; *p* = 4 × 10^−9^). During Fear Generalization, this learning was maintained (i.e., SCRs were significantly greater to the CS+ compared to the CS− during Fear Generalization in both experiments (*p*s < 0.004)).

### Fear generalization: skin conductance responses

**Figure 4:**
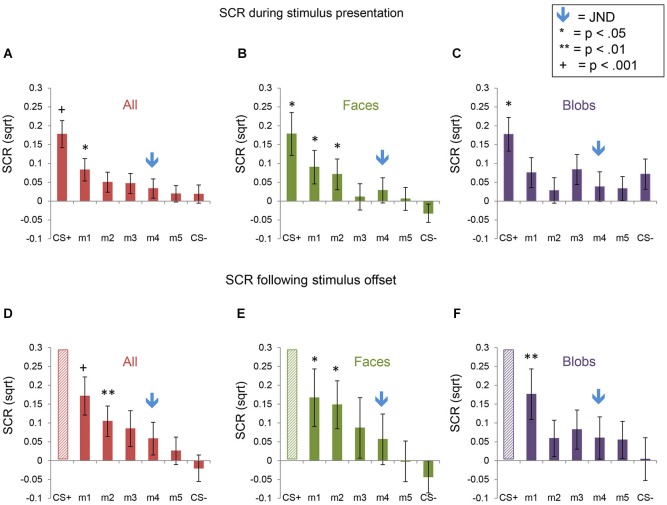
**Skin conductance responses (SCRs) during the fear generalization procedure**. Bar plots of SCRs during fear generalization of the subjects of the two experiments combined (**A,D**; *n* = 47), Experiment 1 (**B,E**; *n* = 24) and Experiment 2 (**C,F**; *n* = 23) are shown. Panels (**A,B** and **C**) show mean maximum SCRs during the 6-s stimulus presentation; panels (**D,E** and **F**) show the mean maximum SCRs following stimulus offset, during the first 6 s of the ITI. Data for the CS+ are omitted from the graphs of the ITI data (panels (**D,E** and **F**), since the responses to the CS+ were likely influenced by the unconditioned stimulus (the electrical shock), which was delivered during the ITI immediately following the presentation of the CS+. A symbol over a CS+, m1 or m2 bar indicates that the mean SCR for this stimulus was significantly greater than the mean SCR to the CS− (* *p* < 0.05; ** *p* < 0.01; ^+^ *p* < 0.001). The blue arrows indicate the morph level corresponding to the JND, m4. Error bars represent one standard error from the mean. Overall, these data reveal that a similar pattern of fear generalization occurs in response to perceptually similar face and non-face control stimuli. In Experiment 1 (faces), there was significant fear generalization to m1 (*t*_(23)_ = 2.44, *p* = 0.02) and m2 (*t*_(23)_ = 2.30, *p* = 0.03) during the stimulus presentation, and to m1 (*t*_(23)_ = 2.55, *p* = 0.02) and m2 (*t*_(23)_ = 2.46, *p* = 0.02) following stimulus offset (*p*s for the other morphs > 0.15). In Experiment 2, there was generalization to m1 only, following stimulus offset (*t*_(22)_ = 2.93, *p* = 0.008), with no significant fear generalization during the stimulus presentation (all other *p*s > 0.08).

Fear generalization was defined by the presence of a significantly greater SCR to a morph (m1 +/− the other morphs) compared to the SCR to the CS− (Lissek et al., 2008; Haddad et al., 2013). The ANOVA revealed a significant effect of stimulus level (*F*_(5,225)_ = 4.78, *p* < 0.001) and a significant stimulus level by experimental phase interaction (*F*_(5,225)_ = 2.55, *p* = 0.03), with no main effects or interactions with stimulus type (all *p*s > 0.22). Follow-up tests revealed that, across both experiments, during the stimulus presentation, there was significant generalization to m1, compared to the CS− (*t*_(46)_ = 2.08, *p* = 0.043; *p*s > 0.25 for the other morph levels). Following stimulus offset, there was generalization to m1 (*t*_(46)_ = 3.80, *p* = 0.0004) and m2 (*t*_(46)_ = 2.74, *p* = 0.009), and a trend towards generalization to m3 (*t*_(46)_ = 1.93, *p* = 0.06) and m4 (*t*_(46)_ = 1.90, *p* = 0.06), with no generalization to m5 (*p* = 0.29) (Figure 4). Thus, there was both immediate (IFG, during stimulus presentation) and delayed (DFG, following stimulus offset) fear generalization to morphs that were perceptually similar to the CS+ (i.e., perceptually closer to and indistinguishable from the CS+, compared to the JND threshold = m4).

**Figure 5:**
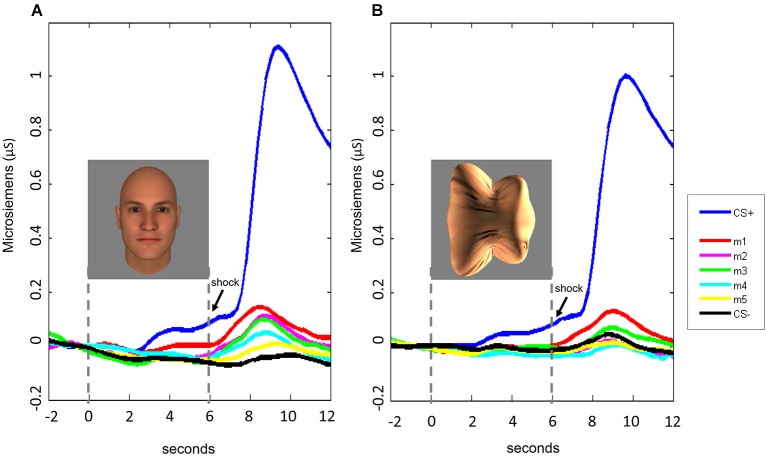
**Time course of SCRs during the fear generalization procedure**. In both Experiment 1 **(A)** and Experiment 2 **(B)**, fear generalization to the morphs was greatest following stimulus offset, peaking at approximately 8–9 s following stimulus onset. For the data displayed here, the baseline was corrected for all stimulus conditions by adjusting the mean response during the 2-s interval before the stimulus onset to 0.

The interaction with experimental phase arose from the greater amount of delayed, compared to immediate, fear generalization (DFG > IFG, Figure 5). A direct comparison of the differential SCRs (response to the morph − the response to the CS−) during the two phases of the experiment confirmed that the responses were greater for m1 (*t*_(46)_ = 3.32, *p* = 0.002) and m2 (*t*_(46)_ = 2.14, *p* = 0.04) (*p*s for the other morph levels >0.12) following stimulus offset, compared to during stimulus presentation.

Consistent with the absence of an interaction with stimulus type in the ANOVA, the pattern of responses was similar across Experiments 1 (faces) and 2 (blobs), although the effects at the individual morph levels appeared to be slightly (but non-significantly) stronger in Experiment 1 (Figures 4 and 5).

### Fear generalization: explicit ratings

**Figure 6:**
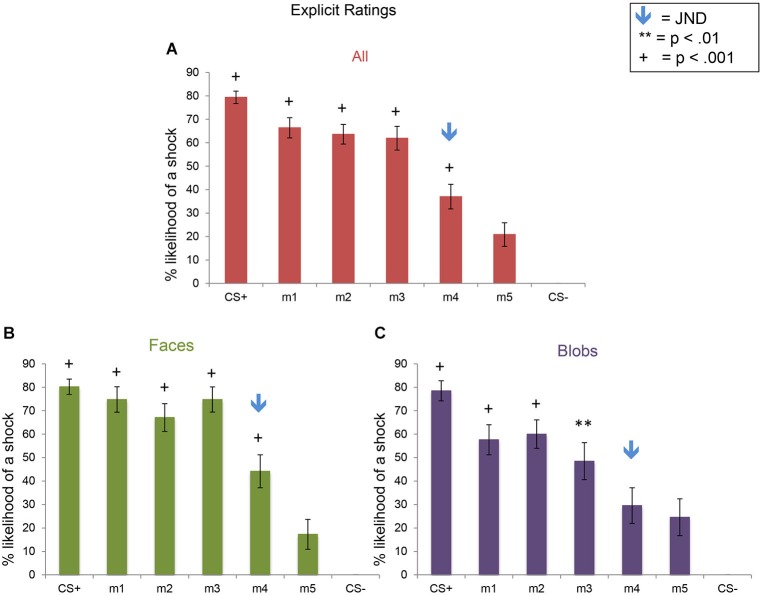
**Explicit shock likelihood ratings following the fear generalization procedure**. Bar plots of shock likelihood ratings for the two experiments combined (**A**; *n* = 47), Experiment 1 (**B**; *n* = 24) and Experiment 2 (**C**; *n* = 23) are shown. A symbol over a bar indicates that the mean ratings for this stimulus were significantly greater than the mean ratings for m5, the morph that was the most different perceptually from the CS+ (** *p* < 0.01; ^+^ *p* < 0.001). The blue arrows indicate the morph level corresponding to the JND, m4. Error bars represent one standard error from the mean. Compared to the m5 ratings, subjects showed significantly greater fear shock likelihood ratings to the CS+ (*t*_(23)_ = 9.00, *p* = 5 × 10^−9^), m1 (*t*_(23)_ = 7.24, *p* = 2 × 10^−7^), m2 (*t*_(23)_ = 7.10, *p* = 3 × 10^−7^), m3 (*t*_(23)_ = 8.12, *p* = 3 × 10^−8^), and m4 (*t*_(23)_ = 3.87, *p* = 0.0008) in Experiment 1 (faces), and to the CS+ (*t*_(22)_ = 5.90, *p* = 6 × 10^−6^), m1 (*t*_(22)_ = 4.43, *p* = 0.0006), m2 (*t*_(22)_ = 4.50, *p* = 0.0002), and m3 (*t*_(22)_ = 2.83, *p* = 0.01), but not m4 (*p* = 0.52) in Experiment 2 (blobs). Direct comparisons of the ratings of the two experiments revealed that there was more explicit fear generalization to faces than to blobs (see text).

For the ERs, there was a significant effect of stimulus level (*F*_(6,270)_ = 61.29, *p* < 0.001), as well as a significant interaction between stimulus type and level (*F*_(6,270)_ = 2.5, *p* = 0.023). This pattern of results arose from the presence of (1) explicit fear generalization to the morph stimuli; and (2) greater fear generalization in Experiment 1 (faces) compared to Experiment 2 (blobs) (Figure 6). Using the CS− as the baseline, comparison condition, we found fear generalization to all morph levels in both experiments (all *p*s < 0.013). However, because the shock likelihood ratings of the CS− were always 0, we also computed fear generalization using the ratings for m5 (the morph that was the most different from the CS+) as the comparison condition. Compared to the m5 ratings, in Experiment 1, subjects showed significantly greater shock likelihood ratings to the CS+, m1, m2, m3 and m4 (all *p*s < 0.0008), whereas in Experiment 2, subjects showed greater shock likelihood ratings to the CS+, m1, m2, m3 (all *p*s < 0.01) but not m4 (*p* = 0.52). Consistent with this, a direct comparison of the ratings across the two experiments at each stimulus level showed that there were significantly higher shock likelihood ratings in Experiment 1 compared to Experiment 2 for m1 (*t*_(45)_ = 2.06, *p* = 0.046) and m3 (*t*_(45)_ = 2.77, *p* = 0.008) (*p*s for the comparisons at the other stimulus levels >0.17).

In summary, although the SCRs showed similar generalization patterns and magnitudes across the two experiments (i.e., to faces and blobs), there was greater *explicit* fear generalization to perceptually similar faces, compared to the non-face control stimuli.

### Direct comparison of autonomic (SCR) and explicit fear generalization in normalized data

#### I. Comparison of shock likelihood ratings vs. SCRs during stimulus presentation

**Figure 7:**
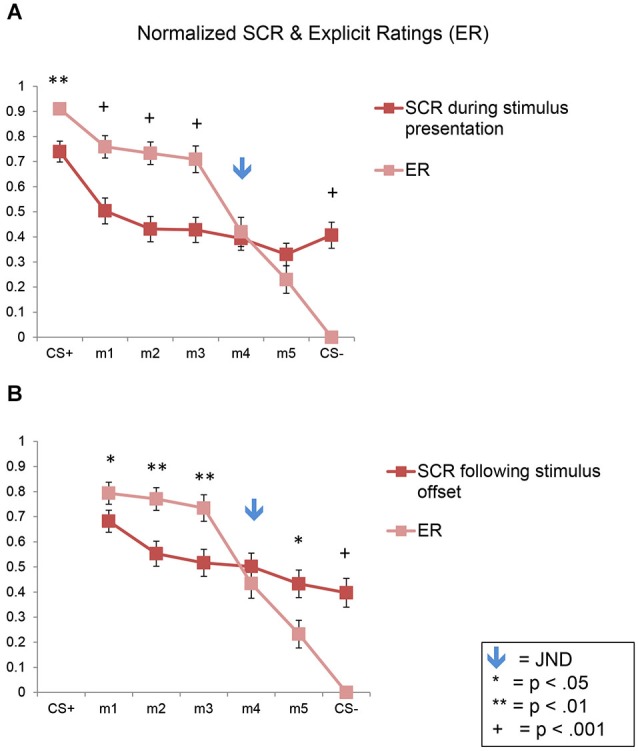
**Direct comparison of SCRs and ERs in normalized data**. Line plots of the normalized SCRs and ratings data for the full sample (*n* = 47). SCR data collected during the stimulus presentation are shown in **(A)**; SCR data collected following stimulus offset are shown in **(B)**. Error bars represent one standard error from the mean. These plots show that conscious fear generalization, as reflected by the explicit shock likelihood ratings, was significantly greater than autonomic, SCR-based fear generalization. The flattening of the SCR plots here (compared to the plots in Figure 3) are due to the normalization process. SCR = skin conductance response; ER = explicit ratings.

Here again we found a significant effect of stimulus level (*F*_(6,270)_ = 46.10, *p* < 0.001), consistent with the results described above showing fear generalization to the morphs for both the SCRs and ERs, across both experiments (Figure 7A). In addition, there was a significant interaction of stimulus level by response type (*F*_(6,270)_ = 17.16, *p* < 0.001), with no significant interactions with stimulus type (*p*s > 0.07). In the normalized data, the ratings values were significantly greater than the SCR values for m1, m2, and m3 (*p*s < 0.002) but not for m4 and m5 (*p*s > 0.21) or CS−, which showed the opposite pattern (*p* = 5 × 10^−10^), since the ratings of the CS− were always 0.

#### II. Comparison of shock likelihood ratings vs. SCRs following stimulus offset

Similar results were found following stimulus offset, with a significant effect of stimulus level (*F*_(5,225)_ = 33.02, *p* < 0.001), and an interaction of stimulus level by response type (*F*_(5,225)_ = 14.49, *p* < 0.001), with no interactions with stimulus type (*p*s > 0.24) (Figure 7B). The ratings values were significantly greater than the SCR values for m1, m2 and m3 (*p*s < 0.05) but not for m4 (*p* = 0.31). Also, m5 and the CS− showed the opposite pattern (*p* = 0.01 and 1 × 10^−8^, respectively).

Thus, this analysis demonstrates statistically that a greater amount of fear generalization was present in the ERs compared to the SCRs in both experiments.

### Correlations between fear learning and fear generalization

In the full sample (*n* = 47), the success of differential fear conditioning (i.e., the magnitude of the difference between SCRs to the CS+ and CS−) predicted the differential SCR to m1 (vs. CS−) during the stimulus presentation (*r* = 0.36, *p* = 0.01) and following stimulus offset (*r* = 0.59, *p* < 0.001), and the differential SCR to m2 following stimulus offset (*r* = 0.48, *p* = 0.001). In Experiment 1 only (*n* = 24), similar correlations were found between fear conditioning success and differential SCRs to m1 during the stimulus presentation (*r* = 0.65, *p* = 0.001) and to m1 and m2 following stimulus offset (m1: *r* = 0.81, *p* < 0.001; m2: *r* = 0.60, *p* = 0.002). Similar correlations were found when the SCRs to the CS− were not subtracted from the SCRs to the morphs.

Fear conditioning success was also correlated with the amount of explicit fear generalization to m1 (ratings to m1 vs. m5) in the full sample (*r* = 0.32, *p* = 0.027, *n* = 47) and in Experiment 2 (*r* = 0.47, *p* = 0.02, *n* = 23).

We found no correlations between JND levels and magnitudes of fear learning or fear generalization.

## Discussion

### Summary of findings

First, we found that fear generalization is closely linked to perceptual discriminability. Specifically, in all analyses, generalization did not occur above discrimination thresholds. Second, we showed that conscious fear responses, measured as shock likelihood ratings, showed a broader fear generalization gradient than the SCRs. Also, both peripheral and conscious measures of fear generalization correlated with the success of acquisition of conditioned fear responses, suggesting that fear generalization here was not due to poor encoding of the original CS-US association. Lastly, partially confirming our prediction, conscious fear generalization was greater in response to faces than to non-face control stimuli.

### Generalization of fear responses is linked to perceptual discriminability

During stimulus presentation, SCR-based fear generalization occurred to the stimulus morph that was perceptually closest to the CS+ (m1), and then extended further following stimulus offset, to include m2 as well. In the ERs, generalization also occurred to m3 and variably (in Experiment 1 only) to m4, which represented the discrimination threshold, but not to m5.

These findings are in line with many previous studies conducted in non-mammalian species (e.g., pigeons responding to varying frequencies of light) showing a relationship between perceptual similarity and generalization of operant responses, which typically have a Gaussian distribution (Ghirlanda and Enquist, 2003). Here, we provide empirical evidence for this type of relationship in humans, demonstrating that the autonomic fear system in humans is sensitive to quite small perceptual differences between stimuli.

The finding of a broader fear generalization gradient in the post-experiment shock likelihood ratings, compared to the SCRs, is consistent with the results of two previous studies that used on-line shock likelihood ratings (Lissek et al., 2008; Haddad et al., 2013), suggesting that this is a robust phenomenon. This dissociation may at first appear counter-intuitive, since the mechanism(s) generating the conscious appraisal of threat seems to be “throwing away” more accurate information possessed by a lower level system.

However, we speculate that this conservative bias in conscious fear responses may have promoted survival during primate evolution. It may be advantageous, in certain contexts, to be wary of stimuli that are similar, but clearly not identical, to known threats, given that these stimuli may have other common characteristics. In the current study, the autonomic system was not mobilized for the morphs that were similar to, but distinguishable from, the CS+, suggesting that the cost of mobilizing the physiological resources to respond to a threat is outweighed, in the short term, by the benefits of gathering more information about the stimulus. A conscious perception of a potential threat may serve the purpose of directing attentional resources towards gathering this additional information (Ledoux, 2000). If new evidence suggests that the stimulus is indeed threatening, then the autonomic fear system may be recruited at that point.

The neural circuitry responsible for these two types of fear generalization responses has not been fully characterized. However it is known that distinct subfields of the hippocampal formation are involved in the individual coding of (vs. the generalization of features across) similar stimuli or events (Aimone et al., 2011; Newman and Hasselmo, 2014). Other studies have reported that the medial prefrontal cortex and midline thalamus also contribute to these processes (Xu et al., 2012; Xu and Südhof, 2013). During face perception, it is likely that the face-selective areas within the ventral temporal cortex, including the fusiform face area (Kanwisher et al., 1997) and anterior temporal area (Rajimehr et al., 2009; Nasr and Tootell, 2012) communicate with this fronto-thalamic-hippocampal memory network.

Consistent with the work conducted in rodents, functional imaging studies of fear generalization in humans using Pavlovian conditioning procedures have found that the medial prefrontal cortex (Dunsmoor et al., 2011; Greenberg et al., 2013a; Lissek et al., 2013a; Cha et al., 2014b) and hippocampus (Lissek et al., 2013a) show response gradients that are consistent with a fear generalization phenomenon. Similar gradients have also been detected in the responses of regions known to be important in salience detection and fear production, such as the insula (Dunsmoor et al., 2011; Greenberg et al., 2013a; Lissek et al., 2013a), striatum (Dunsmoor et al., 2011; Greenberg et al., 2013a) and ventral tegmental area (Cha et al., 2014a). However the mechanisms responsible for integrating the relevant perceptual and motivational information to produce these response gradients remain unclear. Studies that parametrically vary each component (e.g., the perceptual features and motivational value of the stimuli) may clarify how these distinct types of information are used to inform both automatic and conscious perceptions of threat and resulting behavior.

### Autonomic fear generalization has an extended time course

The generalization gradients observed in our SCR data were larger following stimulus offset than during the presentation of the stimulus. This slow time course is typical of SCRs (Bach et al., 2010). This delayed generalization response may also reflect an interaction between the initial autonomic response and the conscious assessment of threat—top-down processes may augment fear responses over time. Alternatively, subjects may experience an acute increase in fear during the time period when they expect to receive a shock, immediately following stimulus offset. The absence of the shock in the context of an increased expectation for it may produce a “prediction error” signal (Li and Mcnally, 2014), contributing to this late SCR. Future studies that manipulate the predictability of the shock may determine whether this response is indeed linked to prediction error-related mechanisms, or merely reflects the long latency of SCRs.

### Conscious fear generalization was greater to faces than to non-face control stimuli

Conscious fear generalization (shock likelihood ratings) was greater to the face stimuli, compared to the perceptually matched control stimuli. Although we can only speculate regarding the mechanisms underlying this effect, one possibility is that the holistic, configural based (vs. feature-based) processing mechanisms relied upon during face perception promotes generalization of fear responses across similar-appearing faces. This hypothesis could be explored further in follow-up work in which, in addition to faces, inverted or contrast-reversed faces (which are processed in a feature-based manner) are used as generalization stimuli.

It is important to also note that our interpretation of this finding is somewhat limited by the fact that we used unrecognizable shapes (“blobs”) as our control stimuli. Fear generalization may be greater for stimuli that are recognizable members of a known category of objects (Dunsmoor et al., 2013) (i.e., clear category membership may facilitate the extraction of general features of objects), compared to stimuli that are unrecognizable and seemingly arbitrary. Future work using non-face, known objects as control stimuli could further test whether fear generalization to faces differs from that to other objects. However, these experiments would also need to account for disadvantages associated with these types of control stimuli, i.e., they would not be closely matched to the face stimuli in terms of lower level cues.

Another open question is whether the pattern of results seen here would change if faces with emotional expressions, such as fear, were used as stimuli. Dunsmoor et al. conducted several studies in which a morph continuum between a fearful and neutral face were used as generalization stimuli (Dunsmoor et al., 2009, 2011). In these experiments, the CS+ stimulus was a morph that was at the midpoint of the fear-to-neutral continuum. They found an asymmetric generalization gradient, with the most fear generalization in response to a morph on the “fear side” of the continuum. Given these data and the results of the current study, one question remains: is there fear generalization to faces with fearful expressions (or other biologically prepared stimuli) that are above the discrimination threshold (i.e., to those that can be clearly discriminated from the CS+) due to their intrinsic aversiveness? An alternative possibility is that discriminability among perceptually similar fearful faces is lower than that to perceptually similar neutral faces (perhaps because of the evolutionary importance of defending oneself rapidly from any possible threat), which would lead to greater generalization across fearful faces. These competing explanations could be investigated with the approach used here in the current study.

### Limitations

This study has several limitations. First, we studied only males, in order to minimize heterogeneity in our data in this first study using this paradigm. A similar study in females is currently underway to determine whether the effects seen here differ across genders. Second, the shock likelihood ratings were not collected during the fear generalization procedure but immediately afterwards. This was done in order to avoid suppression of fear responses by evaluative processes (Lange et al., 2003; Taylor et al., 2003), but this aspect of our design may have affected our results. However, because previous studies that used on-line shock likelihood or fear ratings found qualitatively similar results (i.e., more apparent fear generalization in ratings than in physiological measures) (Lissek et al., 2008; Haddad et al., 2013), this seems unlikely to have had a large effect. Third, our findings could have been influenced by the fact that subjects viewed face or blob stimuli during the discrimination task, before undergoing Pavlovian fear conditioning and generalization procedures with some of the same stimuli. This raises the possibility that other types of learning processes, such as latent inhibition (the inhibitory effect of stimulus pre-exposure on fear conditioning and generalization (Vervliet et al., 2010a)) occurred. However, a latent inhibition effect would have led to a reduction in the level of differential fear conditioning achieved. Given that differential fear conditioning was robust in both experiments, and fear generalization magnitudes correlated with the amount of fear conditioning, this effect was likely small or insignificant.

### Future studies and clinical implications

The development of quantitative measures of perceptual and emotional processes and their interactions is needed for several reasons. After validating such measures, the mechanisms governing these processes can be explored further, by varying the experimental design and measuring additional outcomes, including the underlying brain mechanisms. Also, although some degree of fear generalization is adaptive, excessive generalization of fear or other types of emotional responses may lead to inappropriate behaviors and responses during social interactions, giving rise, in some cases, to psychopathological states. For example, fear generalization has been shown to be excessive in anxiety disorders (Lissek et al., 2010, 2013b; Greenberg et al., 2013b; Kaczkurkin and Lissek, 2013; Cha et al., 2014b). Thus, a quantitative index of abnormal fear generalization may serve as an intermediate phenotype for these disorders, which can serve as a target of treatment and early intervention. Abnormal fear processes have been demonstrated in depression (Nissen et al., 2010) and schizophrenia (Jensen et al., 2008; Holt et al., 2009; Romaniuk et al., 2010) as well. In light of the evidence for abnormalities in neural systems that span diagnostic categories in psychiatry (Insel et al., 2010), the study of fear-related processes in patients with a wide range of symptom types may clarify the degree to which patients with distinct primary diagnoses share a common vulnerability to negative affect and the experience of inappropriate fear.

## Conflict of interest statement

The authors declare that the research was conducted in the absence of any commercial or financial relationships that could be construed as a potential conflict of interest.
